# Evaluation of the effect of a floxed Neo cassette within the dystroglycan (*Dag1*) gene

**DOI:** 10.1186/s13104-017-2926-9

**Published:** 2017-11-21

**Authors:** Francesca Sciandra, Bianca Maria Scicchitano, Giulia Signorino, Maria Giulia Bigotti, Barbara Tavazzi, Francesca Lombardi, Manuela Bozzi, Gigliola Sica, Bruno Giardina, Sandra Blaess, Andrea Brancaccio

**Affiliations:** 1 Istituto di Chimica del Riconoscimento Molecolare (CNR), c/o Università Cattolica del Sacro Cuore, Rome, Italy; 2 Istituto di Istologia, Università Cattolica del Sacro Cuore, Rome, Italy; 3 Istituto di Biochimica e Biochimica Clinica, Università Cattolica del Sacro Cuore, Rome, Italy; 4 School of Biochemistry, University of Bristol, Bristol, BS8 1TD UK; 5 Istituto di Clinica delle Malattie Infettive, Università Cattolica del Sacro Cuore, Rome, Italy; 6 Neurodevelopmental Genetics, Institute of Reconstructive Neurobiology, Life and Brain Center, University of Bonn, Bonn, Germany

**Keywords:** Dystroglycan, Skeletal muscle, Incomplete recombination

## Abstract

**Objective:**

Dystroglycan (DG) is an adhesion complex formed by two subunits, α-DG and β-DG. In skeletal muscle, DG is part of the dystrophin-glycoprotein complex that is crucial for sarcolemma stability and it is involved in a plethora of muscular dystrophy phenotypes. Due to the important role played by DG in skeletal muscle stability as well as in a wide variety of other tissues including brain and the peripheral nervous system, it is essential to investigate its genetic assembly and transcriptional regulation.

**Results:**

Herein, we analyze the effect of the insertion of a floxed neomycin (Neo) cassette within the 3′ portion of the universally conserved IG1-intron of the DG gene (*Dag1*). We analyzed the transcription level of *Dag1* and the expression of the DG protein in skeletal muscle of targeted mice compared to wild-type and we did not find any alterations that might be attributed to the gene targeting. However, we found an increase of the cross-sectional areas of tibialis anterior that might have some physiological significance that needs to be assessed in the future. Moreover, in targeted mice the skeletal muscle morphology and its regeneration capacity after injury did not show any evident alterations. We confirmed that the targeting of *Dag1* with a floxed Neo-cassette did not produce any gross undesired effects.

## Introduction

Dystroglycan (DG) is a widely expressed adhesion complex encoded by a single gene (*Dag1*) as a unique precursor that undergoes post-translational cleavage to generate two interacting subunits: the extracellular and highly glycosylated α-DG and the transmembrane β-DG [1, 2].

DG is crucial for cell membrane stability connecting the actin cytoskeleton to extracellular matrix (ECM) proteins [3]. Whenever the DG/ECM axis is perturbed, severe muscular dystrophy phenotypes do arise [4, 5].

Extensive genetic work has been carried out in order to analyze the function of DG in several tissues [6]. It was shown that the knockout of *Dag1* in mice is embryonic lethal [7]. This result is in line with what was observed in some human patients, in which a homozygous loss-of-function mutation in *Dag1* results in the absence of DG that leads to early postnatal lethality [8].

Conditional knockouts of *Dag1* in several tissues have confirmed the importance of DG for tissue stability. In those studies, no apparent effects of the inserted floxed Neo cassette have been reported [9–11]. Moreover, the knock-in mouse carrying the pathological mutation T190M showed an evident muscular dystrophy phenotype and possible effects arising from the presence of the exogenous genetic elements might have been covered up [12]. On the other hand, in the knock-in mouse Y890F the floxed-Neo cassette had been already removed in the chimeric mice and no effect arising from the presence of the remaining LoxP element has been reported [13].

Interestingly, the insertion of a floxed Neo-cassette can lead to a significantly reduced expression of the target gene and can also downregulate the transcription of genes located at a distance of several kilobases from it [14–22].

Considering the lack of experimental data on the effects of the floxed Neo cassette on *Dag1* expression, we have decided to assess whether the presence of a floxed Neo cassette or single floxed site in the *Dag1* allele might have per se some evident functional consequences for *Dag1* expression in skeletal muscle.

## Main text

### Methods

#### Generation of targeting vector

**Fig. 1:**
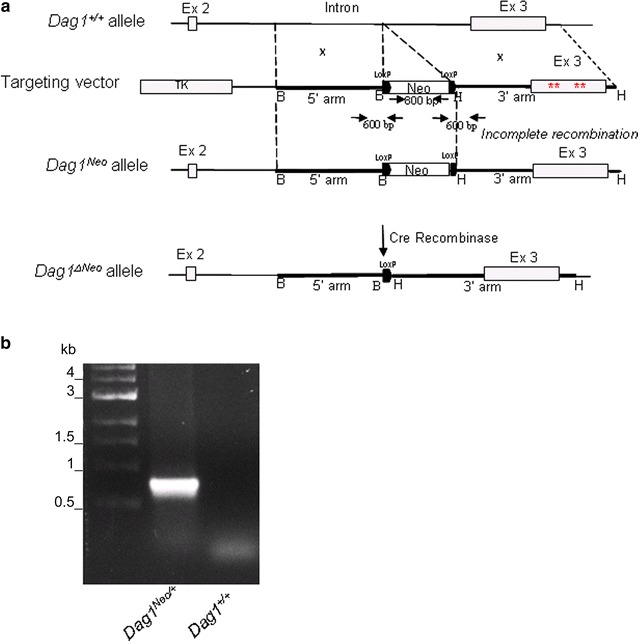
Organization of the dystroglycan gene and position of the floxed Neo cassette. **a** Schematic representation of the *Dag1* ^+/+^ allele, the targeting vector, the *Dag1* allele after incomplete homologous recombination (*Dag1* ^*Neo*^) and the neo-deleted allele (*Dag1* ^*ΔNeo*^). The 5′ arm of the construct consisted of a 3.2 kb fragment amplified from the *Dag1* intron. The 3′ arm was 4 kb in length and consisted of the exon 3′ region, harbouring four mutations (red asterisks) hitting the α/β-DG interface, and flanked by intronic sequences and the 3′-UTR, respectively. A *Xho*I (X) site in the 5′ arm was mutated for cloning purposes. The floxed neomycin cassette (Neo) and the Herpes simplex thymidine kinase (TK) acted as positive and negative selection markers, respectively. Selected restriction enzyme cleavage sites are indicated above the gene (B: *Bam*HI; H: *Hin*dIII and X: *Xho*I). Primers used for genotyping are also indicated as black arrows. **b** A representative PCR genotyping analysis of the *Dag1* ^*Neo/*+^ mouse. The PCR product was amplified from genomic DNA using primers spanning the Neo cassette region

Genomic DNA from V6.4 murine ES (embryonic stem) cells, (genotype C57BL/6J × 129/SvJae), was used as a template to amplify the right and left hand arms of the DG targeting construct. The 5′ arm of the construct consisted of 3.2 kb of intronic sequences amplified from the *Dag1* intron using the following primers: forward 5′-GAGTAAGACTTTGTCTATAAAACA-3′ and reverse 5′-AAACCCCAACAACTACGGTCCTCA-3′. The 3′ arm was 4 kb in length, consisted of the exon 3 region, harboring four mutations, flanked by intronic sequences and the 3′-UTR, respectively, was amplified using the following primers: forward 5′-GAGAAGTGGGATTCATTTAGACAG-3′ and reverse 5′GAATGTAATCTTTAGCTACTGTT-3′. To allow the linearization of the targeting vector that is necessary for the recombination and integration of the construct into the ES cells genome, site-directed mutagenesis was used to mutate a *Xho*I site in the 5′ arm using the QuikChange site-directed mutagenesis kit (Stratagene^®^) and the following primers: forward 5′-AGTTCCTTCCTGCCT**A**GAGTGGGTGTTCCCT-3′ and reverse 5′-AGGGAACACCCACTC**T**AGGCAGGAAGGAACT-3′ (mutated nucleotides in bold). Both the right and left hand arms were initially cloned into separate TOPO vectors (Invitrogen) and then sub-cloned into the final targeting vector, a modified pBluescript KS vector containing a floxed neomycin cassette under the PGK promoter (Fig. 1a). The 5′ and 3′ arms were cloned within a *Bam*HI and *Hin*dIII sites respectively. Both enzymes (New England Biolabs^®^ Inc) were incubated at 37 °C for 1.5 h. The Herpes simplex thymidine kinase (HSVtk) cassette acts as a negative selection marker. The targeting vector was used at the Core Facility for Conditional Mutagenesis, Dibit-San Raffaele Scientific Institute, Milan to electroporate and analyze murine ES cells by Southern blot. One homologous recombinant ES clone was microinjected into blastocysts from 129 J mice. The resulting chimeras were then bred with C57BL6 mice to generate germ line-transmitted heterozygous mice (*Dag1* ^*Neo*/+^). *Dag1* ^*Neo*/+^ mice were backcrossed into the C57BL/6 background for ten generations and then used to create the experimental colonies. Mice from different colonies were crossbred to generate wild-type (*Dag1* ^+*/*+^), heterozygous (*Dag1* ^*Neo/*+^) and homozygous mice (*Dag1* ^*Neo/Neo*^).

To remove the Neo cassette, homozygotes were crossbred with male transgenic mice expressing the transgene Cre under the CMV promoter (Jackson Laboratories) to generate *Dag1* ^*Neo/*+^ mice. Female F1 animals were bred again with homozygous *Dag1* ^*Neo/Neo*^ mice to generate *Dag1* ^*ΔNeo/ΔNeo*^ mice.

#### Creatine kinase assay

Fresh serum was collected by retro-orbital bleeding. The creatine kinase (CK) level was determined according to the International Federation of Clinical Chemistry with commercial reagents on the Cobas 8000/c701^®^ (Roche Diagnostics).

#### Histological and immunofluorescence analysis

All the analyses described below were not performed blind. Tibialis anterior (TA) and gastrocnemius (GA) muscles of 5 months-old female mice (n = 4 per genotype) were embedded in Jung tissue freezing medium and frozen in liquid nitrogen-cooled isopentane. Frozen sections (7 μm) were stained with hematoxylin and eosin (HE) using standard methods [23]. Fiber cross-sectional areas (CSA) of muscle sections were counted manually using ImageJ (NIH, Bethesda, MD) from 6 to 12 randomly selected fields per section. At least 150 fibres per mouse from three mice per genotype were counted.

For the immunofluorescences analysis, TA muscle frozen sections of 5 months-old female mice were incubated with the following antibodies diluted in PBS containing 1% BSA (Sigma-Aldrich): mouse monoclonal anti-α-DG IIH6 (Millipore, code 05–593) (1:100), rabbit polyclonal anti-β-DG (Abcam, code ab43125) (1:300) and rat monoclonal anti-laminin-α2 clone 4H8-2 (Sigma-Aldrich, code L0663) (1:100). The primary antibodies were followed by the appropriate secondary goat anti-mouse IgM (for IIH6), goat anti mouse, anti rat or anti rabbit IgG antibodies conjugated with Alexa^®^488 or Alexa^®^633 fluorophores (1:500) (Invitrogen-Thermo Fisher Scientific). Muscle sections incubated with the only secondary antibody were used as control to validate the primary antibody specificity. Sections were viewed with a Leica SP2 microscope and all images were analyzed using ImageJ software (NIH).

#### Cardiotoxin injury

TA muscles of female 5 months-old mice (four mice per group) were injured along the entire length of the muscle with cardiotoxin (CTX) (LATOXAN SAS, code L8102) injections, 5 μL of 10 μM CTX in saline solution. These conditions ensured muscle damage a uniform manner. The muscles were harvested 10 days after cardiotoxin-induced damage. Frozen sections were processed for histological analysis. Contralateral intact muscles were used as a control. In our previous study, regeneration was fully active at 7 days after injury [24]. Sections were viewed with a Leica SP2 microscope and all images were analyzed using ImageJ software (NIH).

#### Western blotting

TA muscle proteins from 5 months-old mice were extracted with PBS 1% Triton-X100 containing protease inhibitors. 20 μg of soluble proteins were analyzed in Western blot as described elsewhere [24] using the following antibodies: anti mouse α-DG IIH6 (Millipore) (1:1000), anti rabbit β-DG (Abcam) (1:1000) and anti-tubulin HRP-conjugated (Abcam) antibodies.

#### RT-PCR

RNA was isolated from frozen muscle using the RNeasy Fibrous Tissue Mini Kit. Reverse Trascriptase PCR reactions were carried out using 3 μg of RNA. Quantitative RT-PCR was performed in triplicate using a standard TaqMan^®^ PCR protocol on a StepOne Real time PCR System (Applied Biosystems). Probes were specific for mouse *Dag1* and mouse *Gadph* genes. Data analysis was performed as described elsewhere [25].

#### Statistical analysis

Data were expressed as mean ± standard error of the mean (SE). Comparisons among groups were performed using an analysis of variance (One-way ANOVA) followed by a Bonferroni’s post hoc multiple comparison test to determine statistical significance of the differences among the four experimental mice groups; *p* value of < 0.05 was considered to be statistically significant. Statistical analyses were performed with SPSS software (version 17.0, Chicago, IL, US).

### Results and discussion

#### Insertion of the floxed Neo cassette into *Dag1* and removal by Cre recombinase: the *Dag1*^*Neo/Neo*^ and *Dag1*^*ΔNeo/ΔNeo*^ mouse lines

In an attempt to produce a novel DG knock-in mouse line harboring four mutations at the α/β-DG interface [26], an incomplete homologous recombination at the 3′ arm (Fig. 1) prevented the generation of the desired mutated mouse line. Indeed, we generated and studied a mouse line with a *Dag1* allele in which a Neo cassette, flanked by two LoxP sites, was inserted into the second intron (*Dag1* ^*Neo*^ allele) (Fig. 1).

The Neo cassette was introduced in the second intron (also termed IG1-intron since it interrupts the structure of the IG1 domain within the structurally autonomous N-terminal region of α-DG [27]). In addition, we have also crossbred our mice with a mouse line that expresses the Cre recombinase under a CMV-promoter and results in Cre-mediated recombination in germ line. This resulted in the Cre-driven excision of the floxed Neo cassette in the targeted *Dag1* (*Dag1* ^*ΔNeo*^ allele, Fig. 1a, b).

#### Histological and molecular analysis of skeletal muscle

Animals with two wild-type alleles are denoted by “*Dag1* ^+/+^”, mouse lines in which one allele carries the floxed Neo cassette are designated “*Dag1* ^*Neo/*+^”, whilst their homozygous counterpart is denoted by “*Dag1* ^*Neo/Neo*^”. Finally, “*Dag1* ^*ΔNeo/ΔNeo*^” indicates the mouse line obtained upon the excision of the Neo cassette by Cre recombinase.

At 5 months of age, body weights of *Dag1* ^*Neo/*+^ (male 31.5 g ± 2.3; female 25 g ± 1.7), *Dag1* ^*Neo/Neo*^ (male 31.7 g ± 2.6; female 24.5 g ± 1.5) and *Dag1* ^*ΔNeo/ΔNeo*^ (male 31.8 g ± 2.2; female 26 g ± 1.1) mice were not significantly different from wild-type controls (male 31.8 g ± 2.5; female 25 g ± 1.5).

**Fig. 2:**
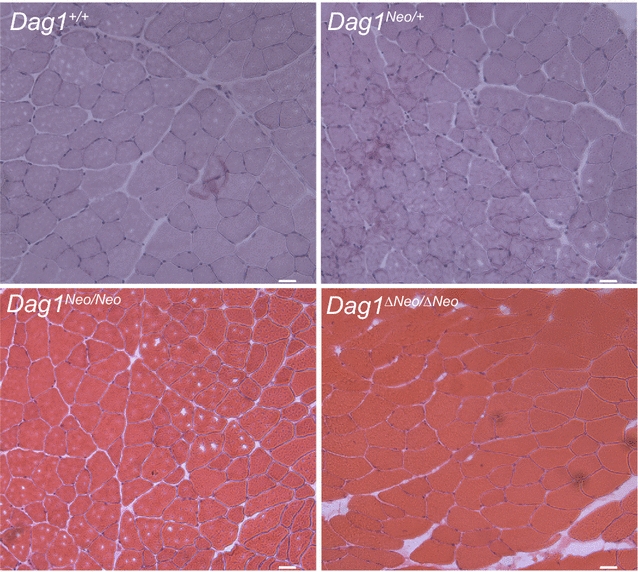
Histological analysis of skeletal muscle. Hematoxylin and eosin staining of transverse frozen sections of tibialis anterior muscle of 5 months old *Dag1* ^+/+^, *Dag1* ^*Neo/*+^, *Dag1* ^*Neo/Neo*^, and *Dag1* ^*ΔNeo/ΔNeo*^ female mice (4 mice per group). No dramatic abnormalities were observed in the skeletal muscle morphology of the targeted mice compared to their wild-type counterparts. Scale bar = 50 μm

**Fig. 3:**
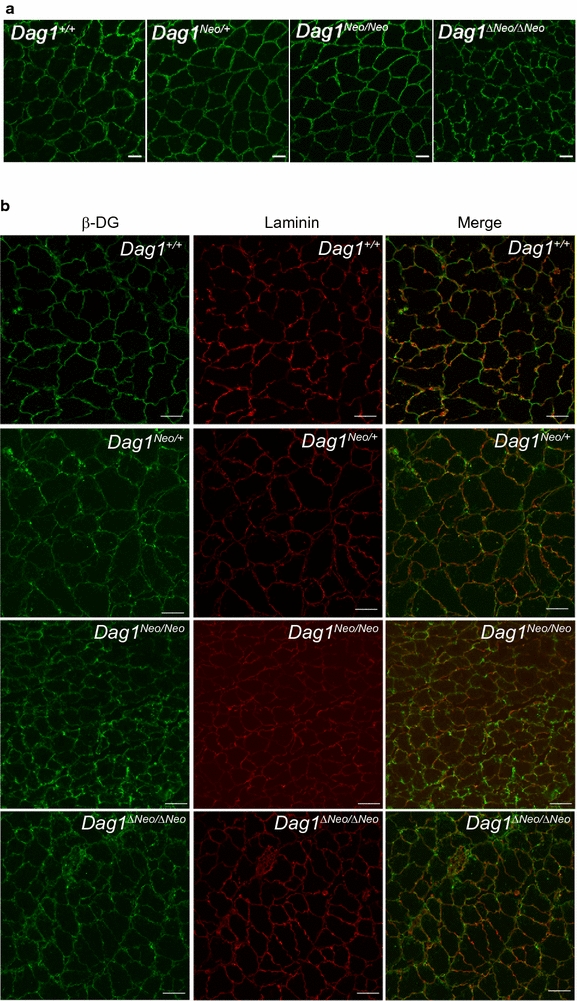
Immunofluorescence staining of skeletal muscle tissue sections. Immunolabeling of **a** α-DG and **b** β-DG and laminin-α2 of 5 months old mice tibialis anterior muscle. Scale bar = 20 μm

**Fig. 4:**
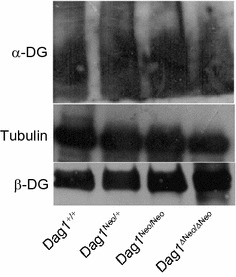
Western blot analysis. Representative immunoblotting of total protein lysates from *Dag1* ^+/+^, *Dag1* ^*Neo/*+^, *Dag1* ^*Neo/Neo*^, and *Dag1* ^*ΔNeo/ΔNeo*^ skeletal muscle. Upper panel; α-DG probed with IIH6 antibody specific for the α-DG carbohydrates epitopes; middle panel: tubulin used as loading control; lower panel: β-DG probed with the anti 43-DAG antibody showing the correct processing of the DG precursor

Histology of tibialis anterior (TA) and gastrocnemius (GA) muscles of 5 months-old female mice, derived from crosses between heterozygotes (intercross) belonging to different colonies, examined by HE staining did not show any gross morphological sign indicating skeletal muscle alterations in *Dag1* ^*Neo/*+^, *Dag1* ^*Neo/Neo*^, and *Dag1* ^*ΔNeo/ΔNeo*^ mice compared to *Dag1* ^+/+^ controls (Fig. 2). In all the *Dag1* targeted mice, the levels of serum CK, a marker for skeletal muscle damage, did not significantly differ from those of wild-type animals (female: *Dag1* ^+/+^ 366 U/L ± 0.8; *Dag1* ^*Neo/*+^ 370 U/L ± 0.8; *Dag1* ^*Neo/Neo*^ 355 U/L ± 0.7; *Dag1* ^*ΔNeo/ΔNeo*^ 360 U/L ± 0.85). CK was measured in 3 and 5-months old mice. Immunofluorescence analysis showed the expected localization of the two DG subunits as well as of the DG major skeletal muscle binding partner laminin to the cell membrane and cell surface, respectively (Fig. 3). Western blot analysis confirmed that the DG precursor is correctly cleaved into its two subunits and that α-DG is highly glycosylated in all the lines (Fig. 4a). The levels of α- and β-DG were similar compared to wild-type mice (data not shown).

**Fig. 5:**
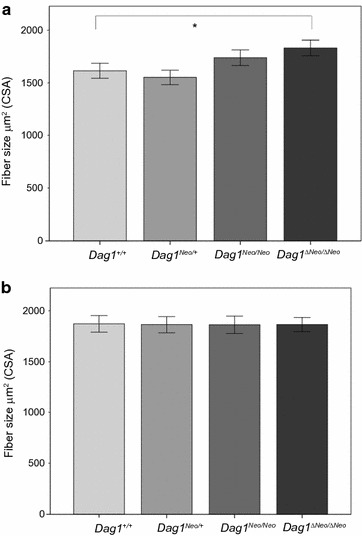
Analysis of skeletal muscle fiber cross-sectional areas (CSA). **a** Tibialis anterior (TA) muscle and **b** gastrocnemius (GA) muscle of *Dag1* ^+/+^, *Dag1* ^*Neo/*+^, *Dag1* ^*Neo/Neo*^, and *Dag1* ^*ΔNeo/ΔNeo*^ 5-months old female mice (4 mice per group). *Indicates significant difference (p < 0.001). Error bars represent SEM

Next, we examined the muscle fiber cross-sectional areas (CSA) by sampling a set of muscle fiber size in TA (Fig. 5a) and GA (Fig. 5b) muscle within the four experimental groups (four mice per group). Overall, we found a significant difference of the medians of fiber CSAs between the groups (lines) of mice (p < 0.001) whereas in GA we did not find any difference (p = 0.998). In particular, in TA muscle the CSA in mouse line *Dag1* ^*ΔNeo/ΔNeo*^ (mean 1826 μm^2^ ± 37) was significantly increased when compared with *Dag1* ^+/+^ mice (mean 1609 μm^2^ ± 36) (p < 0.001). At present, we cannot rule out that the observed variability in TA might have significant physiological repercussions. Further investigations will be required to better clarify this point.

**Fig. 6:**
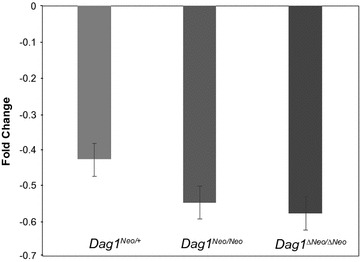
Quantitative analysis of *Dag1* expression in skeletal muscle of 5 months old *Dag1* ^+/+^, *Dag1* ^*Neo/*+^, *Dag1* ^*Neo/Neo*^, and *Dag1* ^*ΔNeo/ΔNeo*^ mice. Total mRNA was purified from tibialis anterior skeletal muscle of 5 months old mice and retro-transcribed to cDNA. Real-Time PCR reactions were performed using probes for the *Dag1* and *Gadph* genes. Results are expressed as fold change of *Dag1* expression in *Dag1* ^*Neo/*+^ (n = 4), *Dag1* ^*Neo/Neo*^ (n = 4), and *Dag1* ^*ΔNeo/ΔNeo*^ (n = 4) lines relative to the *Dag* ^+/+^ line (n = 4). No significant difference was noted between any of the genotypes (p = 0.707). Error bars represent SEM

The increase of TA muscle fibers was not mirrored by an increase either in DG protein levels or in *Dag1* transcription (see below) (Figs. 4, 6). The *Dag1* transcription levels were measured by Real Time PCR experiments that monitor the changes in mRNA levels of *Dag1* ^*Neo/Neo*^, and *Dag1* ^*ΔNeo/ΔNeo*^ mice relative to *Dag1* ^+/+^ littermates and no significant change was detected (p = 0.707) (Fig. 6).

#### Cardiotoxin-induced skeletal muscle regeneration

**Fig. 7:**
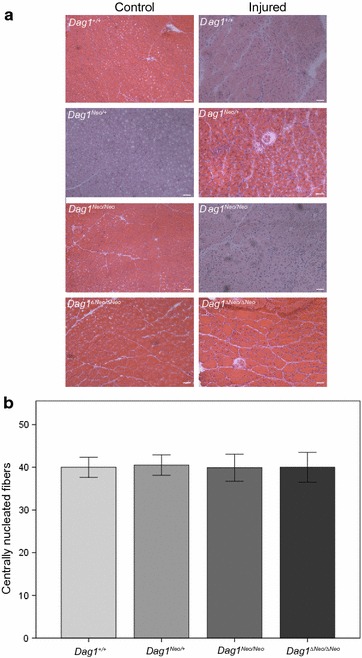
Assessment of skeletal muscle regeneration in *Dag1* ^+/+^, *Dag1* ^*Neo/*+^, *Dag1* ^*Neo/Neo*^, and *Dag1* ^*ΔNeo/ΔNeo*^ mice. Skeletal muscle regeneration process after CTX injection was not affected in *Dag1* ^*Neo/*+^, *Dag1* ^*Neo/Neo*^, and *Dag1* ^*ΔNeo/ΔNeo*^ 5 months old mice compared to *Dag1* ^+/+^ mice. **a** Hematoxylin and eosin staining of transverse frozen sections of tibialis anterior muscle. **b** Quantification of the number of centrally nucleated fibers in mice 10 days after the injection with CTX. Centrally nucleated fibers were counted from five randomly selected fields per section to evaluate fibre regeneration (four mice per group). No significant difference was found among the four experimental groups (p = 0.559). Error bars represent SEM

Dystroglycan is essential for postnatal muscle growth, stability and regeneration [9, 28]. To further analyze the possible presence of skeletal muscle defects of the regenerative process, we analyzed the TA muscles of *Dag1* ^*Neo/*+^, *Dag1* ^*Neo/Neo*^, and *Dag1* ^*ΔNeo/ΔNeo*^ 5-months old female mice compared to *Dag1* ^+*/*+^ mice, 10 days after CTX-induced damage. HE staining revealed that, although CTX injection caused muscle necrosis with the accumulation of mononucleated infiltrating cells, the regeneration response occurred in all the analysed muscles, as demonstrated by the amount of central nucleated fibers present (p = 0.559) (Fig. 7).

## Limitations

The targeting of *Dag1* with a floxed Neo cassette did not produce any strong undesired skeletal muscle phenotype. The increase of CSA that we observed in TA of *Dag1* ^*ΔNeo/ΔNeo*^ mice might be due to some degree of variability between the analyzed mice, but must be further analyzed. *Dag1* second intron is large (10 Kb) and this justifies the absence of relevant effects on *Dag1* mRNA upon the introduction of the floxed Neo-cassette [29].
